# Controlled Release from Zein Matrices: Interplay of Drug Hydrophobicity and pH

**DOI:** 10.1007/s11095-015-1818-8

**Published:** 2015-11-18

**Authors:** Jacob Bouman, Peter Belton, Paul Venema, Erik van der Linden, Renko de Vries, Sheng Qi

**Affiliations:** Laboratory of Physical Chemistry and Soft Matter, Wageningen University, Wageningen, The Netherlands; Laboratory of Physics and Physical Chemistry of Foods, Wageningen University, Wageningen, The Netherlands; School of Pharmacy, University of East Anglia, Norwich, UK; School of Chemistry, University of East Anglia, Norwich, UK; Department of Biomedical Engineering, University of Groningen and University Medical Centre Groningen, Groningen, The Netherlands

**Keywords:** controlled release, diffusion mechanism, dissolution kinetics modelling, extrusion-injection moulding, Zein

## Abstract

**Purpose:**

In earlier studies, the corn protein zein is found to be suitable as a sustained release agent, yet the range of drugs for which zein has been studied remains small. Here, zein is used as a sole excipient for drugs differing in hydrophobicity and isoelectric point: indomethacin, paracetamol and ranitidine.

**Methods:**

Caplets were prepared by hot-melt extrusion (HME) and injection moulding (IM). Each of the three model drugs were tested on two drug loadings in various dissolution media. The physical state of the drug, microstructure and hydration behaviour were investigated to build up understanding for the release behaviour from a zein based matrix for drug delivery.

**Results:**

Drug crystallinity of the caplets increases with drug hydrophobicity. For ranitidine and indomethacin, swelling rates, swelling capacity and release rates were pH dependent as a consequence of the presence of charged groups on the drug molecules. Both hydration rates and release rates could be approached by existing models.

**Conclusion:**

The drug state and pH dependant electrostatic interactions are hypothesised to influence release kinetics. Both factors can potentially be used to influence release kinetics release, thereby broadening the horizon for zein as a tuneable release agent.

**Electronic supplementary material:**

The online version of this article (doi:10.1007/s11095-015-1818-8) contains supplementary material, which is available to authorized users.

## INTRODUCTION

In the field of sustained and controlled release, biopolymers are increasing in popularity as compared to synthetic polymers, since they have the advantages of being biocompatible, environmentally sustainable and often inexpensive. An example is the maize corn protein zein, which has proven to be quite promising for sustained release applications. Indeed, it has been studied for controlled release in formats such as microcapsules (1,2), fibers (3,4), films (5) and monolithic devices (6,7). Sustained drug release is often required to maintain a steady drug plasma concentration for a longer period of time, or to target the drug release to the lower intestinal region in order to maximise absorption. Ideal polymers for sustained release are biocompatible, nontoxic, physically, chemically and microbiologically stable and most important of all, providing a slow but tuneable release (8).

Zein is a major storage protein in maize corn and is a main by-product from bioethanol production by both wet and dry milling (9). It consists of α-, β-, γ- and δ-zein, which are proteins with different molecular weights and modes of extraction (10,11). The α-zein is the predominant protein whose structure is a triple superhelix (12). The yellow colour of the powder is due to the lutein, localised in the non-polar interior of this helix. Possibly due to this structure, zein is relatively heat and pH stable (13), water insoluble, but soluble in aqueous ethanol (14). The protein has hydrophobic and hydrophilic domains, but due to its insolubility in water, it is frequently considered to be a hydrophobic protein (15). However, the hydrated protein is found to absorb more water upon heating indicating a hydrophilic nature (14). A similar conclusion is based on the calculated hydration energy of the protein on the basis of calculated hydration energies (14) The combination of these characteristics and its biocompatibility (15) and anti-oxidative activity (16) make it a good candidate as sustained release matrix material.

The use of zein micro- and nanoparticles as drug delivery devices (produced by the use of an anti-solvent precipitation process) has been reported by a number of groups (1,2,17). In these studies, *in vitro* sustained drug release profiles were reported. However, in the presence of pepsin, quick degradation of zein occurs (1), leading to equally quick disintegration and high drug release rates. The use of electrospinning and electro-spraying to create zein nanofibers and nanoparticles have also been reported (3,4,15). For these systems there are problems in maintaining the stability of the fiber- and particle morphologies that at this stage still need to be solved by crosslinking the zein molecules (15). An interesting observation made in these papers is that electrostatic interactions, between the drug and the zein matrix, contribute significantly to the compatibility between the drug and zein (15,18).

The preferred and most widely used mode of drug delivery is still oral delivery in the form of tablets. Some work has already been performed on macroscopic zein based devices for oral administration (6,7,19). The advantage of using zein for macro scale devices is that the effect of degradation of the zein network by pepsin, will be much less of a problem in engineering sustained release since at the macroscale, hydration of zein by itself already takes hours (6). Previously zein has been investigated as a tableting excipient for the sustained release of theophilline. It was found that compression of the zein led to high elastic recovery after pressing and resulted in unacceptable grooving inside of the tablets (7). To bypass such problems, we have recently investigated the use of zein as a hot-melt extrusion-injection moulded (HME-IM) caplet excipient (6). After HME-IM, zein caplets loaded with a model drug (paracetamol) showed a close to diffusion-controlled sustained release: the release could be precisely tuned *via* the device dimensions, and was found to be nearly independent of drug loading.

Paracetamol is a moderately hydrophilic, uncharged model drug. In order to be useful as a general excipient it is crucial to understand how factors, such as, drug hydrophobicity and the presence or absence of charged groups on the drugs, affect the interaction with the zein matrix and hence, the release profile. Therefore, we study the drug release behaviour of HME-IM zein caplets for a range of model drugs. In addition to paracetamol, we also investigate the highly hydrophilic drug ranitidine (which is positively charged at low pH) and the hydrophobic drug indomethacin (which is negatively charged at high pH). Zein has an isoelectric point (pI) around neutral pH values. Therefore we expect to find differences in release rates as a function of pH, caused by changes in the electrostatic interactions of the drugs with the zein matrix. This is the first report on the influence of drug hydrophobicity and pH on the mechanism of drug release from macroscopic zein matrices.

First we use DSC and PXRD to characterise the caplets in terms of drug crystallinity. X-ray tomography (XRT) is used together with scanning electron microscopy (SEM) to investigate the microstructures of the caplets before and during the drug release process. Finally, we study both the swelling- and drug release kinetics of the caplets, for a range of pH values.

## MATERIALS AND METHODS

### Materials

Purified zein protein flakes were obtained from Acros organics (Geel, Belgium). In order to decrease particle size to ensure good mixing with the different crystalline drugs, it was ground using a kitchen blender. The grinding process consisted of cycles of 15 s grinding followed by a 20s of cooling, to minimise thermal damage to the protein as a result of frictional forces. Grounded zein powder particle size was roughly between 50–250 μm as estimated from microscopy images. The drugs used, *i.e*., paracetamol, indomethacin and ranitidine, were all obtained from Sigma Aldrich (Gillingham, UK).

### Extrusion and Injection Moulding

Distilled water (10–12% of weight mixture) was mixed with the ground zein powder in a glass mortar and pestle, to lower the glass transition temperature (T_g_) of zein, so hot-melt extrusion could be performed at a relatively low temperature. Drug was added to the zein-water mixture to obtain the mixes for formulations with drug loading of 4.4 and 22.2% (w/w). The ternary mixes (zein-water-model drug) were ground again prior to extrusion. A batch of 5–10 g was processed using a HAAKE™ Minilab extruder (Thermo Fisher, Karlsruhe, Germany) with a co-rotating twin screw. Extrusion temperature was 80°C, the screw speed was 100 rpm and no die was used. Extrudates were collected in a pre-heated injection moulding cylinder (80°C) which was attached to the extruder. Also the IM process was performed at 80°C, under high pressure of 300–350 bar, where the still flexible extrudate was pressed into a caplet mould using a HAAKE™ MiniJet Pro Piston Injection Moulding System (Thermo Fisher, Karlsruhe, Germany). After the IM sequence, the mould was placed on aluminium foil positioned on dry ice for a rapid cooling. The caplet (5.9x4.4x19.7 mm) was ejected out of the mould and stored in a sealed container at ambient conditions.

### Differential Scanning Calorimetry (DSC)

DSC experiments were conducted using a Q2000 MTDSC (TA Instruments, Newcastle, DE, U.S.). A full range calibration was performed prior to sample testing. For the DSC samples, a caplet slice was cut and milled into smaller particles using mortar and pestle. Prior to the DSC measurements, all samples were dried in a desiccator above phosphorus pentoxide, for 4 days at room temperature, to minimize the effect of water evaporation during a DSC run. A standard heat-cool-heat sequence was applied for all samples, with 10.00°C/min heating rate and 5.00°C/min cooling rate. In the DSC cell a nitrogen purge at a flow rate of 50 mL/min was used. Analysis was performed using TA Universal Analysis software. To measure the amount of crystalline drug present, melting enthalpies were compared with the melting enthalpy of pure crystalline drug. Melting enthalpies for pure indomethacin, paracetamol and ranitidine were experimentally measured at respectively 98.0 ± 1.1 J/g, 181.0 ± 0.7 and 130.0 ± 2.1 J/g. For ranitidine melting is quickly followed by degradation. Therefore the apparent melting enthalpy may be heating rate dependent but, provided the rate is kept constant in all the experiments amounts of crystalline material may be estimated. The DSC measurement is only semi-quantitative as there may be dissolution of the drug during the heating process and the calculation neglects any contribution from the entropy of dissolution of the drug after melting. However as the mixture is unstirred during the heating process dissolution before melting is likely to be very small. Similar a significant error due to dissolution after melting would require a rapid diffusion of the drug away from its location and into the matrix which is not likely. Apparent melting enthalpy may be heating rate dependent but, provided the rate is kept constant in all the experiments a semi-quantitative estimate of crystalline material may be made.

### X-ray Tomography (XRT)

XRT images were taken using a Phoenix v[tome]x m (General Electric, Wunstorf, Germany). This technique allows non-invasive measurement of the 3D structure of objects at spatial resolution below 1 μm. A 3D image of a fixed particle can be reconstructed from a large series of two-dimensional radiographic images taken around a single axis of rotation (20). The following settings were applied: voxel size: 4 μm, amount of images: 2000, Voltage 100 kV, Current 110 μA and timing 500: ms

### Powder X-Ray Diffraction (PXRD)

PXRD measurements were performed on caplets and pure powders using a Thermo Scientific™ ARL™ X’TRA Powder Diffractometer (Ecublens, Switzerland) fitted with a Cu (copper) x-ray tube. The following operating conditions were used: current, 40 mA; voltage, 45 kV; step size, 0.01° and acquisition time of 1.0 s/step.

### Scanning Electron Microscopy (SEM)

Caplet pieces, cut using a razor blade, were attached to sample holders with carbon adhesive tabs (EMS, Washington, USA) and sputter coated with 10 nm Iridium (SCD 500, Leica, Vienna, Austria). Hereafter they were analyzed with secondary electron detection at 2 KV and 6.3 pA with a high resolution scanning electron microscope (Magellan 400, FEI, Eindhoven, the Netherlands).

### Dissolution and Hydration Media

Media with 4 different pH’s were prepared. The pH 1 solution was a 0.1 M HCl solution. The pH 5.5 medium was a sodium acetate-acetic acid buffer. Both the pH 6.8 and pH 7.8 media were phosphate buffers prepared from potassium dihydrogen phosphate and sodium hydroxide.

### Hydration Studies

Hydration was measured gravimetrically. Caplets (diameter 5.9 mm) were carefully cut into slices (thicknesses was 2.20 ± 0.03 mm) by the use of a razor blade. Slice thicknesses and weight were measured respectively using an electronic calliper and a microbalance. Caplets were immersed in a medium solution at 37°C with 50 rpm paddle rotation speed. At regular time intervals, the samples were removed from the dissolution bath. Excess water was gently removed using a piece of paper towel before weighing the samples. Subsequently the samples were immediately returned to the solution. The weight change of the samples was monitored over a period up to 70 h. The water uptake *M* (*g*_*water*_/*g*_*zein*_) was determined in 2 steps. First the initial sample weight minus the weight of the released drug (based on the Peppas fits on the data obtained from the release experiments (see Fig. 5 and Table II), was subtracted from the measured wet weight of the sample. Then, the obtained increased weight was divided by the dry weight of the zein in the sample. All hydration experiments were performed in triplicate.

### Dissolution Studies

Dissolution experiments were performed using the paddle method with 900 ml of dissolution media at 37.0 ± 0.5°C and a 50 rpm rotation speed of the paddles. Caplets were carefully cut into slices, which were measured and weight as in the hydration experiments. Release kinetics was measured at regular time intervals. 10 ml samples were taken and measured using a UV/vis spectrophotometer (S-22 Boeco, Boeckel and Co., Hamburg, Germany) at 243 and 314 nm corresponding to the λmax of respectively paracetamol and ranitidine. Because indomethacin has a low saturation concentration in water, a biphasic dissolution method needed to be applied on indomethacin loaded caplets, to keep the chemical potential difference between the sample and the dissolution medium constant. Caplets were immersed in 600 ml medium, while on top a 300 ml octanol phase was present. Release was measured in the octanol phase by UV/vis at 319 nm, corresponding to a λ_max_ of indomethacin. In dissolution media where pH is above pK_a_ (which is around 4.2), indomethacin is negatively charged, which could decrease uptake in octanol and theoretically disrupt the test. However when testing the release of drug crystals at pH of 6.8, it was found to be very fast compared to other pH’s as can be seen in Fig. 9c. Therefore we have no reason to believe the charge of the drug will be disruptive for the test.

## RESULTS AND DISCUSSION

### Drug-Zein Compatibility

To assess the compatibility between zein and the model drugs, it is not possible to use theoretical Flory-Huggins based drug-excipient miscibility prediction methods, such as ground contribution and solubility parameters methods (21), as zein protein has a complex structure. A first indicator for the drug-matrix compatibility is the influence of drug loading on the processability during hot-melt extrusion-injection moulding (HME-IM), as reflected by the extrusion torque. A higher torque indicates poorer zein-drug miscibility or poorer plasticisation of the zein matrix by the drug.

Our results for the extrusion torque for the model drugs (at two different drug loading) are shown in Table I. We the found that loading the zein matrices with the hydrophilic drug ranitidine reduces the extrusion torque. On the other hand, loading the matrix with the hydrophobic drug indomethacin increases the torque. For paracetamol we find an intermediate result, with little or no changes in the torque compared to the unloaded zein. As we will discuss more extensively in a later section, the changes in torque reflect the zein-drug miscibility at the temperatures at which extrusion occurs. We verified that small variation in moisture content (0.12–0.14 (w/w)) did not influence the torque values during extrusion (data not shown).

**Table I:** Average Extrusion Torque for Each Drug Loading. Extrusion Torque Increase with Drug Hydrophobicity. Paracetamol Extrusion Torque is Most Similar to that of Unloaded Zein

	Average extrusion torque (Ncm)			
Drug loading	Zein	Indomethacin	Paracetamol	Ranitidine
No drug	30			
4.4% drug		40	30	20
22.2% drug		45	30	20

**Fig. 1:**
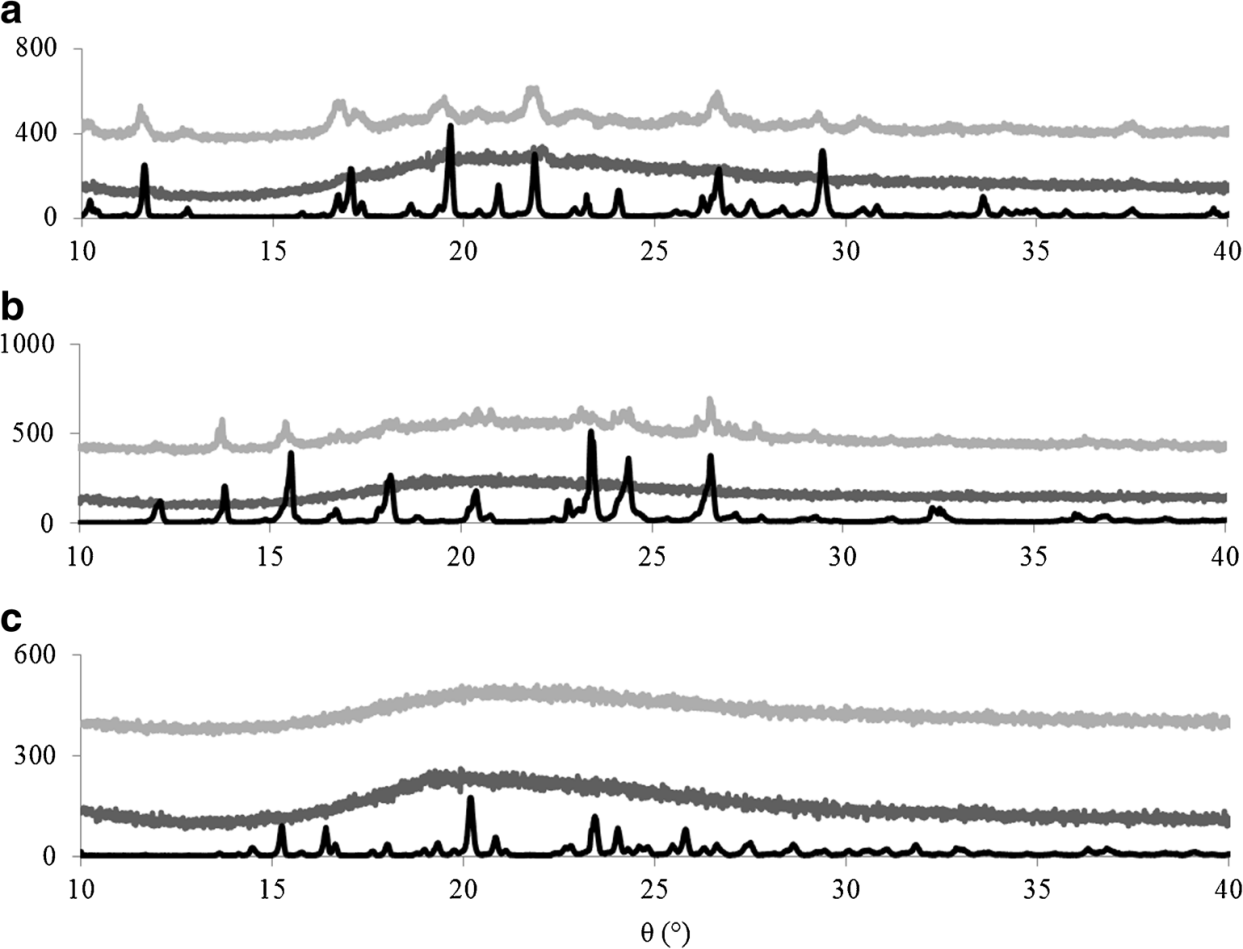
PXRD results of caplets containing different drug loadings (%), indicating crystallinity of the drug in the different caplets. Drugs loadings are 22.2%() and 4.4%() are displayed together with pure drug (). Drugs used are (**a**) Indomethacin, (**b**) paracetamol and (**c**) ranitidine. Results show increase in crystallinity with drug hydrophobicity in the HME-IM processed caplets.

The degree of crystallinity of the drugs in the caplets was determined using both PXRD and DSC. PXRD results are shown in Fig. 1. For the most hydrophobic drug, indomethacin, we find some degree of crystallinity of the drugs in the caplets for both drug loadings (4.4% and 22.2%). For the paracetamol, only the caplets with a drug loading of 22.2% have some degree of crystallinity, in agreement with our previously published work (6). For the most hydrophilic drug, ranitidine, we find no indications of crystalline drug present in any of the caplets.

**Fig. 2:**
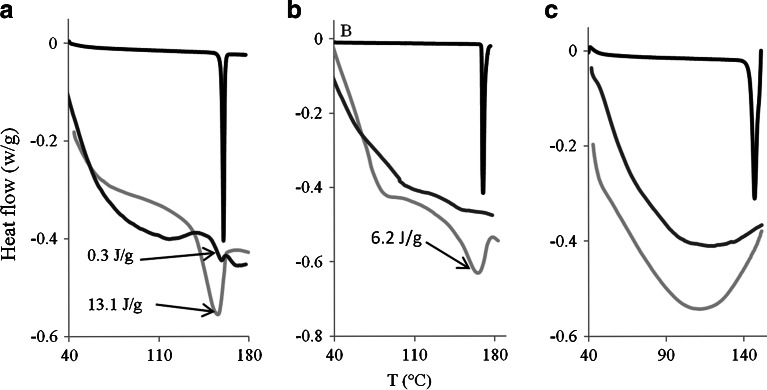
DSC thermograms of the zein/drug caplets containing different drug loadings: 22.2% () and 4.4%(). Heat flow (W/g) is plotted vs Temperature T (°C). Drugs are (**a**) Indomethacin, (**b**) paracetamol and (**c**) ranitidine. A reduced curve () of the pure drug is added as comparison. Melting enthalpy is displayed for caplets if melting peak was present.

The corresponding DSC data is shown in Fig. 2 and confirms the PXRD results. In addition, for the case of indomethacin, we use the DSC data to quantify the fraction of drug in a crystalline state, from the melting enthalpy of the crystalline drug melting peaks. This estimate is only semi-quantitative, as discussed above. We find that at 4.4% loading, the estimated degree of crystallinity is 0.3%. This increases to 13% at the higher drug loading of 22.2%.

Overall, our PXRD and DSC data show that the degree of crystallinity increases with hydrophobicity of the drug and also with drug loading. The degree of crystallinity is in line with the differences we observe in the HME processability of the three model drugs. The HME-IM temperature was set at 80°C which is still significantly below (70–90°C) the melting points of the drugs. Crystals which remained undissolved during HME could act as a filler (22) and this can explain the increase in extrusion torque for indomethacin. For the case of ranitidine, for which extrusion torques are lowest of all used mixes, we believe that the drug acts as a plasticizer of the zein. Note that the loss of crystallinity during extrusion is significant for all of the drugs we tested. We believe this cannot be caused by dissolution of the drug in the small amount of water used during the pre-mixing stage of the sample preparation, since the saturation concentrations of indomethacin, paracetamol and ranitidine around pH = 6.5 are respectively 0.33 g/L (23), 17.4 g/L (24) and 660 g/L (25). Given the water contents of the mixes (which are below 0.14 w/w), the theoretical maximal dissolution of drugs in water at 20°C would be, respectively 3.9°10^−3^, 0.2 and 7%. The increased solubility at the temperatures of extrusion (80°C) are also insufficient to explain the large decrease of the degree of crystallinity. For instance, we estimate that the theoretical maximal dissolution of paracetamol in the water present in the caplet at 80°C, can increase to only 1.5% at most, based upon extrapolation of published solubility curves (26).

Therefore, the most likely explanation is that the zein/water matrix ‘solubilizes’ the drugs during extrusion. The amount of drug that is dispersed in the matrix may be expected to correlate inversely with the drug hydrophobicity. Nevertheless, even the hydrophobic indomethacin appears to be solubilized to a significant extent, as reflected by the large decrease of the degree of crystallinity upon incorporating the indomethacin in the zein matrix *via* HME. Therefore, we conclude that zein, processed using HME, is efficient at bringing a range of drugs into a non-crystalline state, which may be beneficial for drug delivery applications. This will be especially important when the dissolution of the drug crystals is slower than desired.

### Microstructures of the Drug-Loaded Caplets

**Fig. 3:**
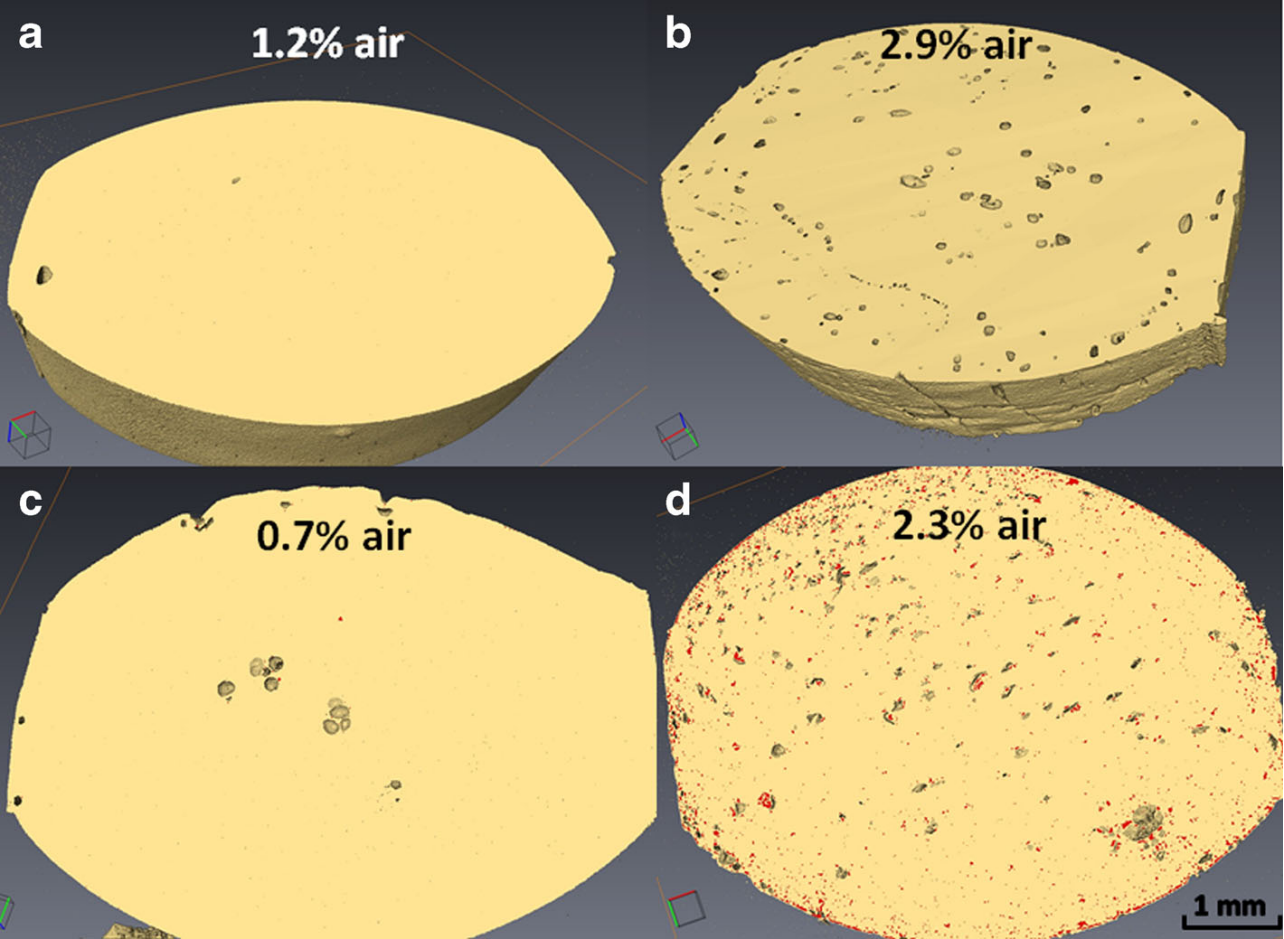
XRT images of caplets, showing presence and distribution of denser (drug) domain (in red) together with volume for air pockets. (**a**) unloaded zein, (**b**) 22.2% ranitidine, (**c**) 22.2 paracetamol and (**b**) 22.2% indomethacin. Volume of air pockets (%) is displayed for each caplet.

We first investigate the structure of the drug-loaded caplets on the micrometer length scale, using XRT. Figure 3 shows XRT images (with a resolution of around 5 μm) of an unloaded zein caplet and of 3 zein caplets loaded with 22.2% of each of the three model drugs. For the caplets containing indomethacin and paracetamol, we observed domains with a significantly higher density than the background (indicated in red in Fig. 3). For indomethacin the volume percentage of these domains is 9.1%, while for paracetamol the volume percentage of these domains was only 2.1%. In both samples the domains are largely homogeneously distributed in the bulk of the caplet. For indomethacin caplets, it appears that there is a higher concentration of such domains close to the surface of the caplets. We found that during processing some air was included in the caplets, leading to some degree of porosity. Differences in porosity could influence the rate of hydration, but also the release rate as hypothesised in an earlier study on zein membranes (5). From the estimated volume percentage of pores (see Fig. 3), the ranitidine and indomethacin loaded samples are found to have a higher volume percentage of pores than the paracetamol loaded and unloaded zein caplets. For all measured systems, the overall volume fraction of the pores is in the order of a few percent.

**Fig. 4:**
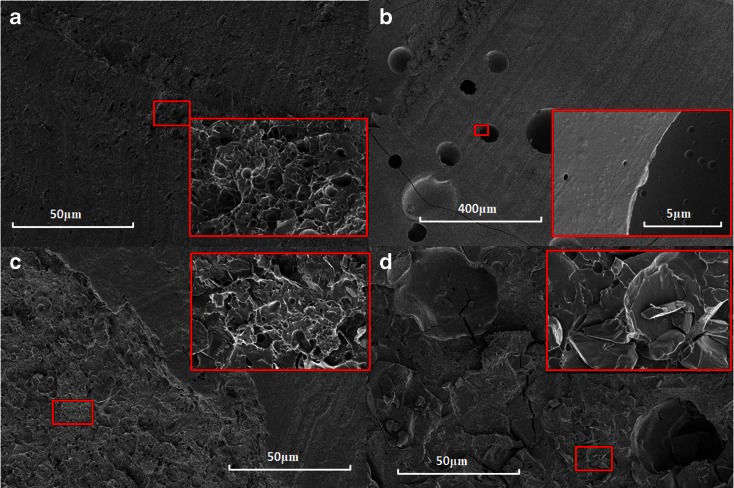
SEM images of drug-loaded zein caplet cross sections. (**a**) unloaded zein, (**b**) 22.2% ranitidine, (**c**) 22.2% paracetamol and (**d**) 22.2% indomethacin. The insert of each picture shows a zoom of the area displayed by the smaller red box.

Next we used SEM to investigate the same microstructures at an even higher resolution. Figure 4 shows representative SEM images for an unloaded zein caplet and for caplets loaded with 22.2% of each of the three model drugs. The images for the paracetamol loaded caplets are similar to those for the unloaded zein caplets. According to the SEM images, caplets with ranitidine appear to have a significantly more smooth surface compared to the other caplets. Finally, for indomethacin loaded caplets, the SEM images clearly show the presence of distinct particle-like structures, that could be the dispersed indomethacin crystals. The SEM images also demonstrate the presence of pores at smaller length scales than those probed by XRT. Hence the actual porosity of the caplets may be somewhat higher than the values estimated on the basis of XRT (see Fig. 3).

An important observation is that the volume percentage of the domains with a higher electron density in XRT (shown in red in Fig. 3) is similar to the degree of crystallinity as estimated on the basis of the DSC results. This strongly suggests that the dense domains in the XRT images can be identified as the drug crystals dispersed in a zein-drug matrix. The SEM images also confirm that only in the indomethacin caplets a significant fraction of crystals is present. It was also observed that many of these crystals are located in the pores of the caplet caused by the incorporation of a small fraction of air during the processing, as shown clearly in supplementary materials Figure S1.

The porosity due to the air incorporated in the caplets, was found to be higher for indomethacin and ranitidine than for the paracetamol and unloaded caplets. This may or may not be related to the fact that both indomethacin and ranitidine strongly influenced the extrusion torque. Even though the actual volume fraction of air that causes porosity is not high, the differences in porosity that we observe should be born in mind, in view of the large effect that such pores were suggested to have on release hydration and release properties .

### Hydration and Swelling Kinetics

**Fig. 5:**
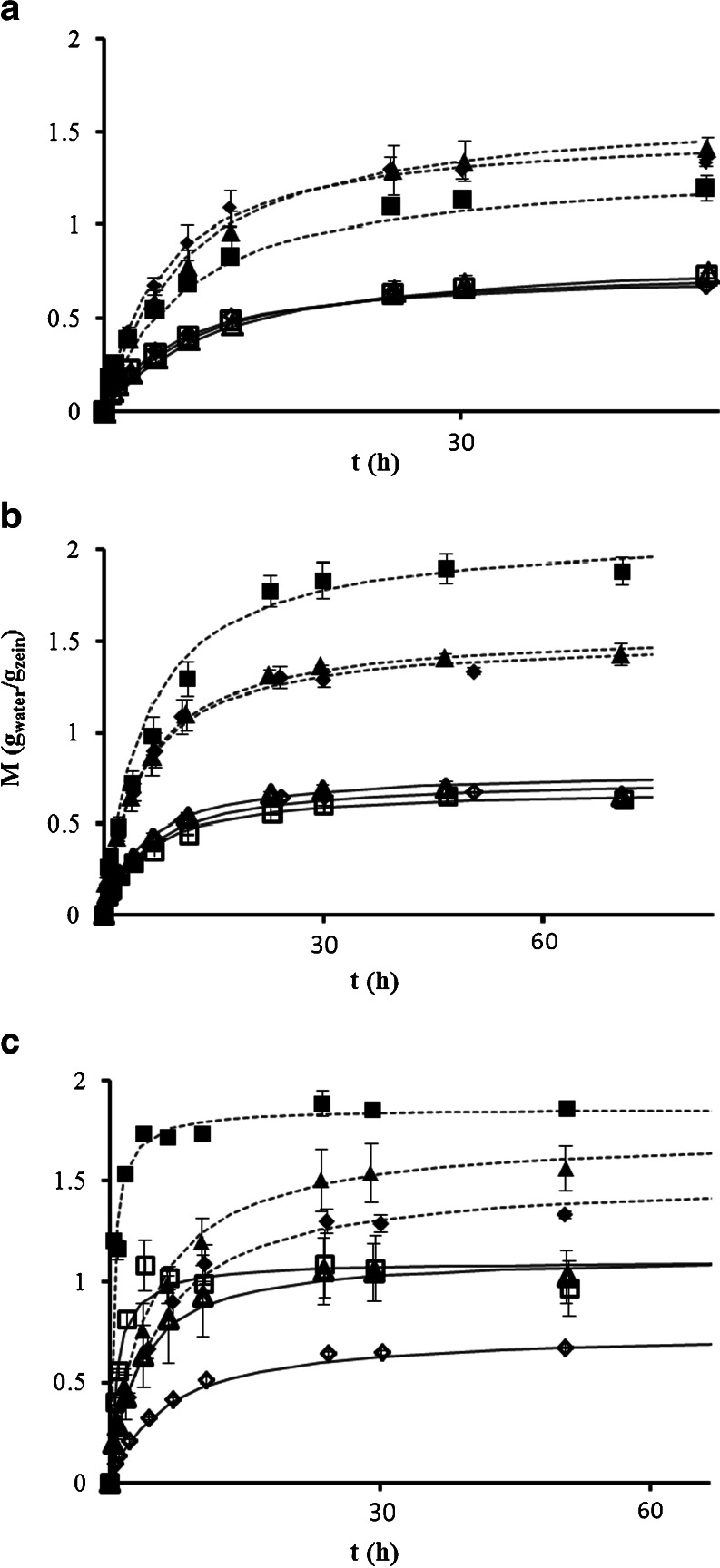
The water uptake *M* (*g*_*water*_/*g*_*zein*_) as a function of time t (*h*) for the different caplets used. Drug loadings at 4.4% (▲,△), 22.2% (■,□) and no (♦,◇) drug loading in pH 1 (▲,■,◆) and pH 6.8 (△,□,◇). The drugs tested are (**a**) indomethacin, (**b**) paracetamol and (**c**) ranitidine. In all graphs, the water uptake curve of non-loaded zein caplets is added as comparison. The lines are fits using the Peleg model, Eq 1, to caplets in pH 1 (*continious line*) and pH 6.8 (*dashed line*) medium.

The hydration and swelling behaviour is expected to influence the release behaviour of matrix-based delivery systems. Water uptake of all samples was measured in two different dissolution media, HCl solution (pH 1) and phosphate buffer (pH 6.8) (see Fig. 5), which were also used in the dissolution experiments. The data was fitted to the Peleg (27) equation, which is widely used as empirical equation to fit water uptake of matrices:

1 $$ M(t)-{M}_0=\frac{t}{K_1+{K}_2t} $$

where *M*(*t*) is the moisture content (*g*_*water*_/*g*_*zein*_) at time *t* (*min*), *M*_0_ is the initial moisture content, *K*_1_ is the Peleg rate constant (*min*∙(*g*_*water*_/*g*_*zein*_)^−1^) and *K*_2_ is the Peleg capacity constant (*g*_*water*_/*g*_*zein*_)^−1^. The values of the fitting parameters 1/*K*_1_ and 1/*K*_2_ for the different caplets are shown in Table II.

**Table II:** Fitting Parameters of the Caplet Hydration Data of A) Unloaded Caplets and b) Caplets with Different Drug and Drug Loadings in Different Media. For Fitting the Peleg Equation (Eq 1) was Used, Where 1/*K*_*1*_ Displays Initial Hydration Rate and 1/*K*_*2*_ is a Measure of Swelling Capacity

A									
				Zein					
	$$ \begin{array}{c}\hfill 1{0}^2\cdot 1/{K}_1\hfill \\ {}\hfill \frac{g/g\; zein}{min}\hfill \end{array} $$			$$ \begin{array}{c}\hfill 1/{K}_2\hfill \\ {}\hfill g/g\; zein\hfill \end{array} $$			R_2_		
pH = 1	0.52			1.52			0.996		
pH = 6.8	0.21			0.75			0.997		
B									
	Indomethacin			Paracetamol			Ranitine		
Drug loading, pH	$$ \begin{array}{c}\hfill {10}^2\cdot 1/{K}_1\hfill \\ {}\hfill \frac{g/g\; zein}{min}\hfill \end{array} $$	$$ \begin{array}{c}\hfill 1/{K}_2\hfill \\ {}\hfill g/g\; zein\hfill \end{array} $$	R^2^	$$ \begin{array}{c}\hfill {10}^2\cdot 1/{K}_1\hfill \\ {}\hfill \frac{g/g\; zein}{min}\hfill \end{array} $$	$$ \begin{array}{c}\hfill 1/{K}_2\hfill \\ {}\hfill g/g\; zein\hfill \end{array} $$	R^2^	$$ \begin{array}{c}\hfill {10}^2\cdot 1/{K}_1\hfill \\ {}\hfill \frac{g/g\; zein}{min}\hfill \end{array} $$	$$ \begin{array}{c}\hfill 1/{K}_2\hfill \\ {}\hfill g/g\; zein\hfill \end{array} $$	R^2^
4.4%, pH = 1	0.41	1.64	0.998	0.52	1.56	0.998	0.71	1.74	0.995
4.4%, pH = 6.8	0.15	0.85	0.996	0.25	0.79	0.994	0.64	1.13	0.994
22.2%, pH = 1	0.32	1.32	0.996	0.50	1.89	0.990	8.33	1.86	0.982
22.2%, pH = 6.8	0.18	0.79	0.997	0.21	0.69	0.990	2.33	1.10	0.956

For all caplets, both the hydration rates (1/*K*_*1*_) and the swelling capacity values (1/*K*_*2*_) are much higher at pH 1 as compared to the values at pH 6.8. Hydration rates (1/*K*_*1*_) of paracetamol caplets are comparable to those of unloaded zein caplets, while hydration rates (1/*K*_*1*_) of indomethacin caplets are slightly lower and decrease with drug loading. For 22.2% loaded ranitidine caplets we find hydration rates that are much higher than those of unloaded caplets (~16.5 times higher at pH 1 and about 11 times higher at pH 6.8). As for the dependence of the swelling capacity (1/*K*_*2*_) on the drug loading ratio, we find qualitatively different behaviour at pH 1 and pH 6.8. At pH 1 an increased drug loading has an increasing (for paracetamol and ranitidine) or decreasing (for indomethacin) effect on the swelling capacity. In contrast, at pH 6.8 caplets loaded with indomethacin and paracetamol show very similar swelling capacity as compared to values for the unloaded zein caplets. This is not the case for ranitidine caplets since they show an increase of 1/*K*_*2*_ at pH 6.8 compared to unloaded zein caplets.

The influence of pH on both hydration rate and swelling capacity can be related to the charge of the zein protein. The zein predominantly consists of α-zein, which has an isoelectric point of pI 6.8, hence at pH 1 zein is positively charged and at pH 6.8 it will be uncharged. As a result, at pH 1 the proteins will have more counter ions present which increases the osmotic pressure in the caplet and cause an increase in swelling (28). Another possible contribution to faster swelling at pH 1 may be the de-amidation of glutamine and asparagine amino acids of the zein (29), which may also lead to a more open network. At identical drug loadings, both hydration rate and swelling capacity decrease with drug hydrophobicity, since the addition of the hydrophobic drugs to the zein matrix simply makes the combined system more hydrophobic. Probably, once the hydration takes place, the drug dissolution leads to an decrease of the chemical potential of the water in the matrix resulting in the uptake of water by the matrix.

**Fig. 6:**
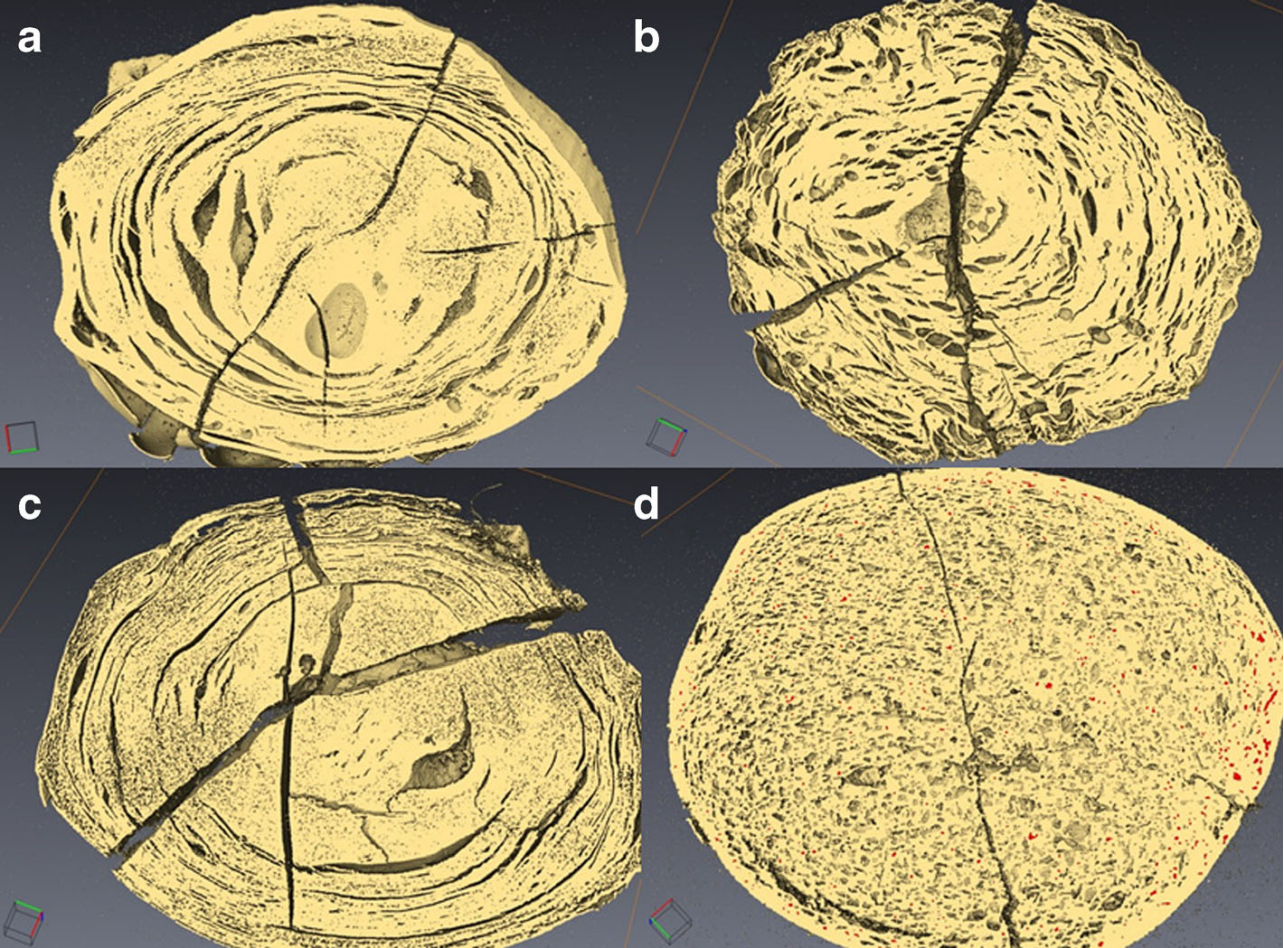
XRT pictures of caplets hydrated for 96 h at pH 1 and subsequently vitrified and freeze dried. The zein caplets were loaded as follows: (**a**): no loading, (**b**): 22.2% rantidine, (**c**):22.2% paracetamol, (**d**): 22.2% indomethacin. Radial cracks are most likely caused by the vitrification process.

**Fig. 7:**
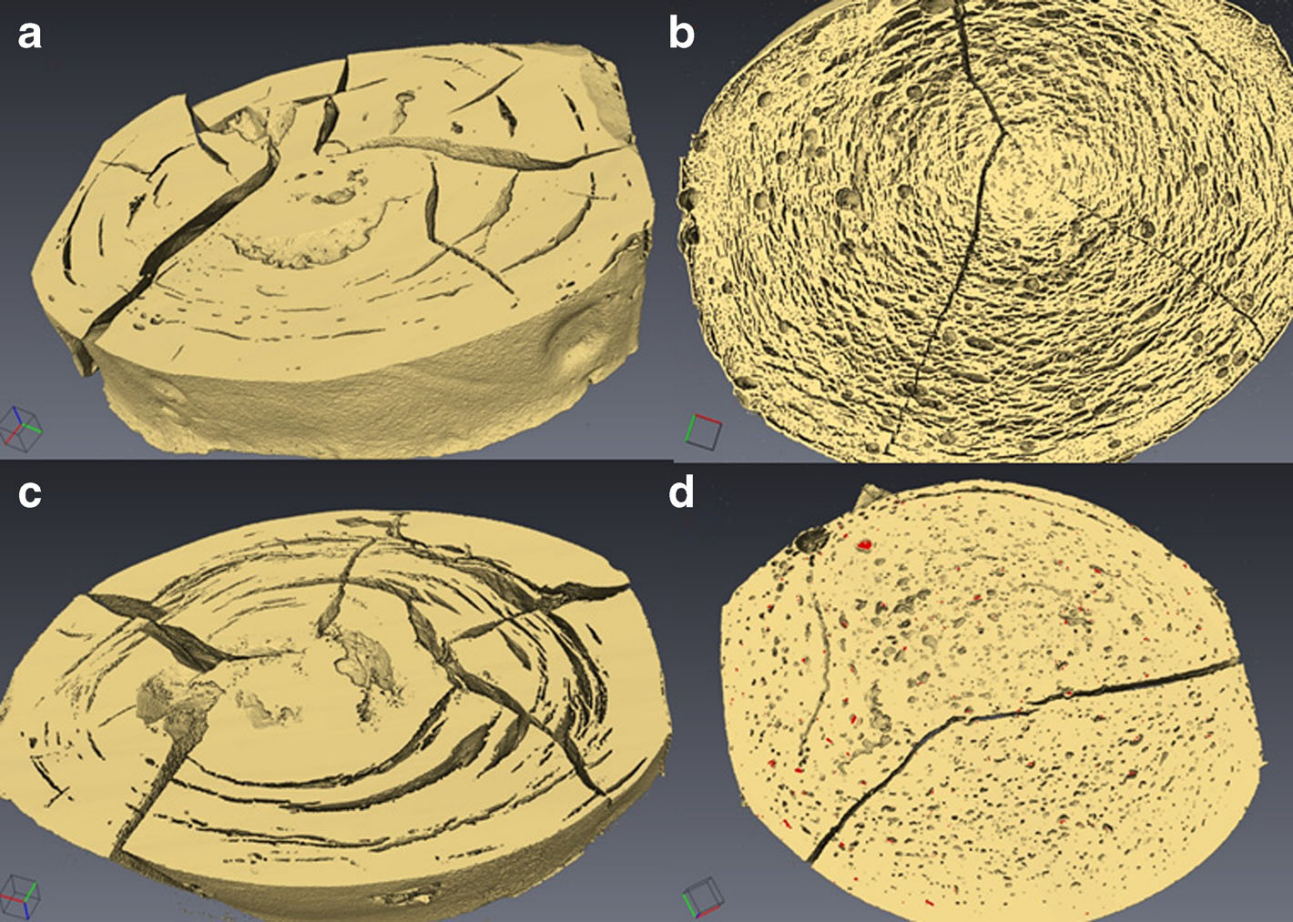
XRT pictures of caplets hydrated for 96 h at pH 6.8 and subsequently vitrified and freeze dried. The zein caplets were loaded as follows: (**a**): no loading, (**b**): 22.2% rantidine, (**b**):22.2% paracetamol, (**d**): 22.2% indomethacin. Radial cracks are most likely caused by the vitrification process.

**Fig. 8:**
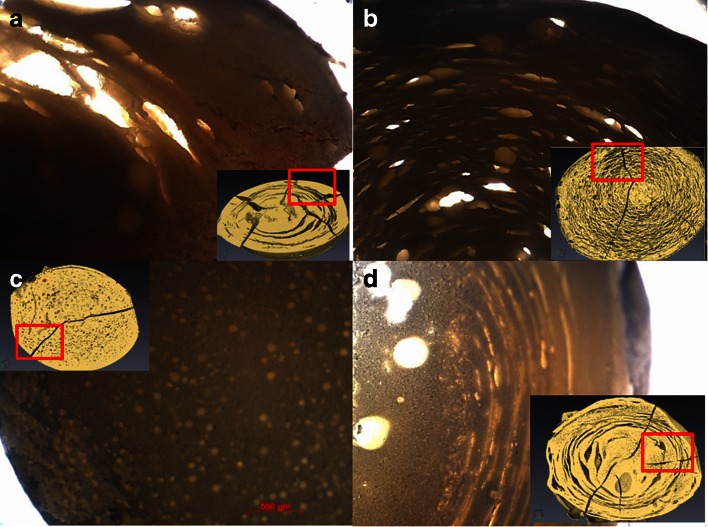
Light microscopy image of (untreated) hydrated caplet slices compared with XRT of vitrified and freeze dried hydrated caplet (insert). (**a**) 22.2% paracetamol pH = 6.8 (**b**) 22.2% ranitidine pH = 6.8 (**c**) 22.2% indomethacin pH = 6.8 (**d**) zein pH = 1

Next, the dynamic changes in the microstructure of the caplets caused by hydration and swelling were studied in more detail using XRT. After 96 h of hydration, the caplets were rapidly vitrified in liquid nitrogen and subsequently freeze-dried. XRT images of caplets that were hydrated at pH 1 and pH 6.8 are shown in Figs. 6 and 7. The similarity of the XRT images of the freeze-dried caplets and the light microscopy images of wet hydrated caplets (Fig. 8), confirmed that the freeze-drying process did not affect most of the interior microstructures of the caplets, with the exception of the formation of the radial cracks.

In both Figs. 6 and 7, it can be observed that the zein and paracetamol-loaded caplets, show pronounced concentric circular cracks after hydration, similar to tree rings. For the ranitidine and indomethacin-loaded caplets, the cracks are much less pronounced. In the ranitidine sample the concentric circular cracks are still visible, while in the indomethacin sample they are only visible at the outer region close to the surface of the caplet. Previously, it was suggested that the cracks are a result of stresses on the network due to hydration (30). The correlation that we find between on the one hand hydration rates and capacities (Table II) and on the other hand the occurrence of the circular cracks (Figs. 6 and 7) is therefore expected. The circularity of the cracks may be explained if we assume that hydration is controlled by case II diffusion (6). Since for case II diffusion there is a sharp boundary between the hydrated and unhydrated parts of the caplet, rupture is expected to take place at this boundary, that migrates inwards driven by the hydration process. For the ranitidine caplets the concentric cracks are much finer. This could be a consequence of the higher fraction of air and associated porosity of the ranitidine caplets. The pores might relieve some of the stress due to the passing hydration front, leading to a finer crack structure.

### Drug Release

**Fig. 9:**
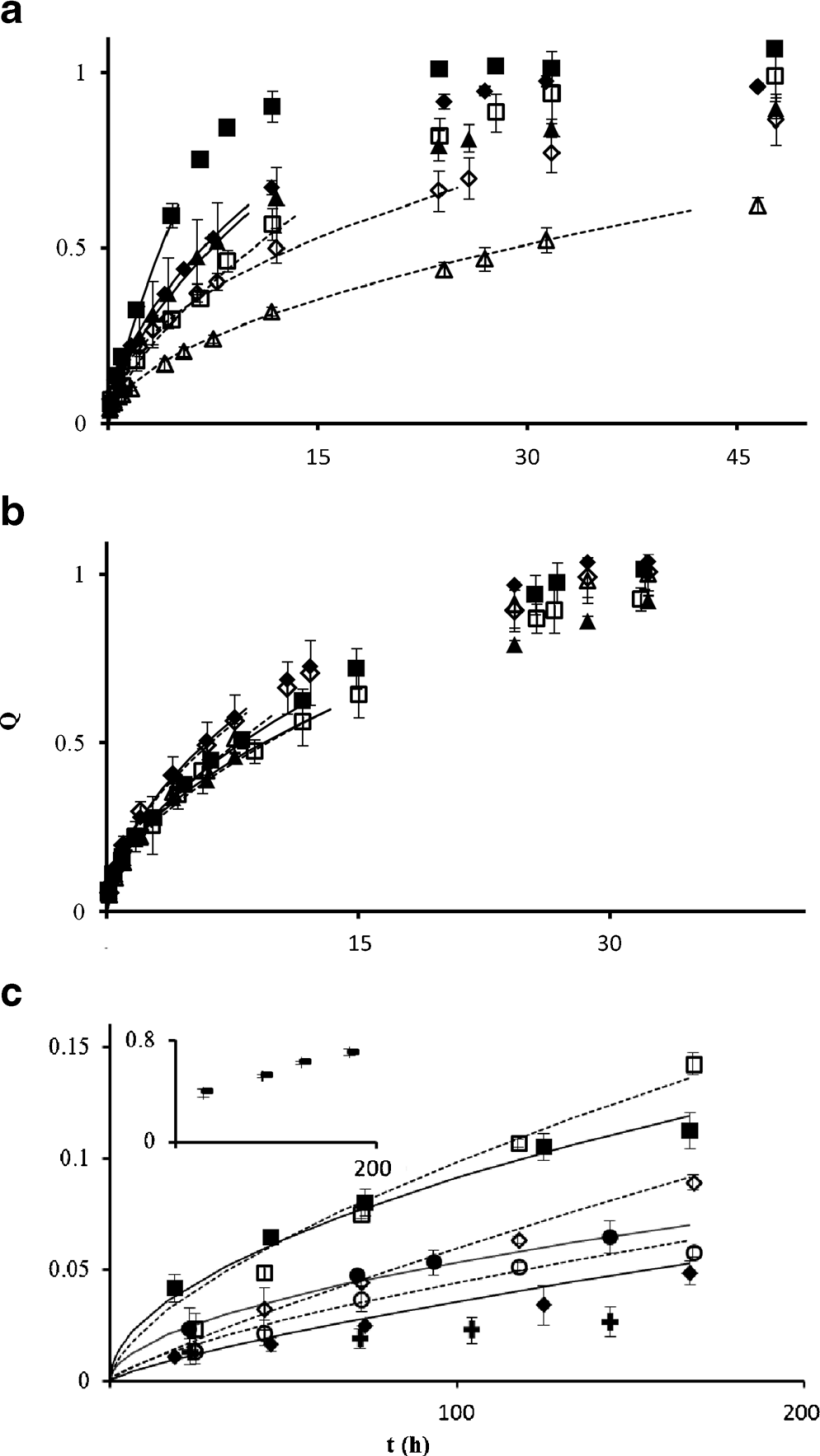
Release profiles for slices of caplets at pH 1 (◇, ◆), pH 5.5 (○,●), pH 6.8 (■, □) and pH 7.8 (▲, △). The caplets were loaded with (**a**) ranitidine, (**b**) paracetamol and (**c**) indomethacin, with a 4.4% loading (△,□,◇,○) and a 22.2% loading (▲,■,◆,●). The lines show data fits using the Peppas model, given by Eq. 2., to caplets with a 4.4% drug loading (dashed line) and a 22.2% drug loading (continuois line). In (**c**), release from pure indomathcin crystals shown at pH 1 (+) and pH 6.8 (−) (see insert).

Release profiles for paracetamol and ranitidine loaded caplets were measured by the conventional dissolution method, while for the caplets containing the poorly soluble indomethacin, the biphasic dissolution method was used (31). Here, an octanol phase was added to maintain sink conditions in the aqueous phase throughout the dissolution test. The indomethacin that is dissolved in the aqueous phase, is expected to be efficiently extracted by the octanol. For each drug an additional medium (with pH 7.8 or pH 5.5) was tested to further investigate the influence of pH on release kinetics. Results are shown in Fig. 9 and also shown are fits of the release profiles to the Peppas equation (32):

2 $$ \frac{Q(t)}{Q_{\infty }}=k{t}^n $$

Here *Q* is the mass of drug released at time *t* (minutes) and *Q*_∞_ is the mass of drug release as time approaches infinity. The parameter *k* depends on the characteristics of the excipient-drug network, while *n* is an exponent indicative for the transport mechanism. For the cylindrical geometry of the caplets with a slice thickness-to-diameter ratio of 2.7, two limited mechanisms can be identified. When the release kinetics is controlled by pure Fickian diffusion (driven by a chemical potential gradient (33)), the expected value is *n* = 0.45. On other hand, when release is controlled by Case II diffusion (commonly associated with a glass-to-gel transition of the matrix, for which the matrix hydration step is controlling the release rate (33)), the expected value is *n* ~ 0.9. The same value for *n* may also be found for very poorly soluble drugs, when release is limited by the dissolution rate of the drug. Values for the parameters *k* and *n* determined by fitting the Peppas model to the experimentally observed release kinetics for the three model drugs (up to a released fraction of 0.6) are given in Table III. For ranitidine and paracetamol caplets, values for the half-life (indicative for the typical release time) are also given in Table III.

**Table III:** Parameters Describing the Kinetics of Release for the Different Caplets Containing Paracetamol, Ranitidine or Indomethacin. R^2^ Values of the Fits Were Between 0.992 and 1.000. For Indomethacin Linear Fits Were Applied Giving a Slope and Intercept

Drug loading, pH	Ranitidine			Paracetamol			Indomethacin		
	k∙10^2^	n	half-life (min)	k∙10^2^	N	Half-life (min)	k∙10^5^	n	Half-life (min∙10^−4^)
4.4% pH 1	2.16 ± 0.45	0.47 ± 0.04	761	1.93 ± 0.27	0.55 ± 0.03	358	4 ± 0.6	0.84 ± 0.04	7
22.2% pH 1	1.73 ± 0.15	0.56 ± 0.01	416	1.97 ± 0.07	0.55 ± 0.01	354	4 ± 2	0.78 ± 0.05	18
4.4% pH 5.5	–	–	–	–	–	–	10 ± 5	0.70 ± 0.05	19
22.2% pH 5.5	–	–	–	–	–	–	53 ± 30	0.53 ± 0.07	41
4.4% pH 6.8	0.67 ± 0.07	0.67 ± 0.07	600	1.98 ± 0.65	0.51 ± 0.06	553	41 ± 8	0.63 ± 0.05	7
22.2% pH 6.8	0.77 ± 0.03	0.77 ± 0.01	221	1.67 ± 0.17	0.55 ± 0.02	472	99 ± 60	0.52 ± 0.06	16
4.4% pH 7.8	0.96 ± 0.10	0.53 ± 0.02	1819	1.11 ± 0.08	0.62 ± 0.03	452	–	–	–
22.2% pH 7.8	1.21 ± 0.35	0.61 ± 0.07	454	1.62 ± 0.11	0.54 ± 0.02	594	–	–	–

A first observation is that for most paracetamol and ranitidine caplets, the exponent *n* is between 0.45 and 0.65, while for indomethacin caplets, the value of *n* is on average 15% higher. For paracetamol loaded caplets the release kinetics is almost independent of drug loading and release medium, in line with earlier results (6). For ranitidine, release rates drop significantly at the highest pH (pH 7.8), but increase with drug loading. With the exception of 22.2% loaded caplets at pH 6.8 and pH 7.8, the ranitidine caplets appeared to show slower release rates compared to paracetamol caplets. Not unexpectedly, indomethacin caplets showed a much slower release as compared to caplets containing paracetamol and ranitidine. The release rate for the indomethacin caplets was highest at pH 6.8, and lowest at more acidic pH values (lowest at pH 5.5 for 4.4% caplets and lowest at pH 1 for 22.2% caplets). Except at pH 1, the release kinetics was found to be quite insensitive to drug loading. The release kinetics of the paracetamol and ranitidine are found to be close to Fickian diffusion. The values of *n* that we find for indomethacin, at pH 1 indicate that here the limiting step is the dissolution of amorphous or crystalline drug particles. The solubility of indomethacin is known to be strongly pH dependent (23) being less than 2.7 mg/L at pH 1. We have also tested release rates for indomethacin crystals ourselves and compared with the release rates from caplets, as shown in Fig. 9. In the experiment, the crystals were kept in dialysis tubes to prevent their migration to the octanol phase. We confirmed that while at pH 6.8, release rates from crystals is much faster than from the caplets. For pH = 1 it is just the other way around, suggesting that in the latter case the caplet preparation procedure has molecularly dissolved at least part of the drug into the matrix leading to faster release than from crystals.

**Fig. 10:**
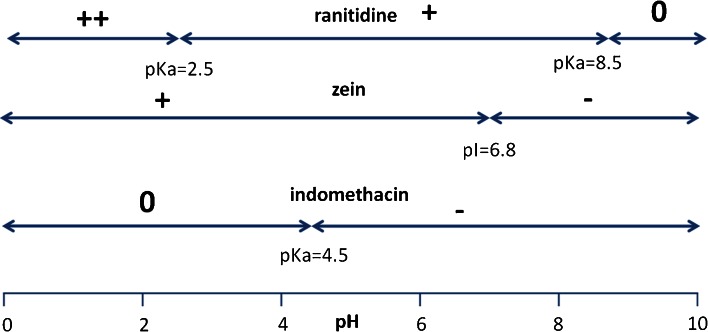
Schematic overview of the charge of ranitidine, zein and indomethacin at different pH’s. pKa and pI values for the compounds are also shown. Electrostatic attractions are higher between ranitidine and zein between pH 6.8 and pH 8.5 and higher between indomethacin and zein between pH 4.5 and 6.8.

A key factor influencing the pH dependence of the release kinetics is obviously the electrostatic interaction between the drugs and the zein matrix. In Fig. 10 the pI of zein and the pKa values of ranitidine and indomethacin are displayed together with their resulting charge at different pH values. Paracetamol is not included, because it is uncharged in all test media (34). If charges on the drug and the matrix have opposite signs, there is an electrostatic attraction and the release rate may be expected to become lower. Indeed, this is what is observed for ranitidine. For this case we find a significant difference in release rates between the case of pH 6.8 (where zein has no net charge) and pH 7.8 (where zein is has a net positive charge), especially for 4.4% loaded caplets. Likewise, for indomethacin one would expect the slowest release between pH 4.5 and pH 6.8 where the drug and matrix carry opposite charge signs. Indeed, for 4.4% loaded caplet we find that release at pH 5.5 is even slower than at pH 1.

The hydration rate and swelling capacity do not seem to directly correlate with the release kinetics. As shown in Fig. 5, hydration rate and swelling capacity are much higher at pH 1 than at pH 6.8. However, for paracetamol caplets, release is only slightly faster at pH 1 than at pH 6.8. For ranitidine release is even slower at pH 1 than at pH 6.8. The water solubility of the drug does not seem to be the main determinant of release kinetics either, since release of paracetamol from the caplets was in most cases faster than the release of the much more soluble ranitidine (20 times more soluble). For the very poorly soluble indomethacin, solubility does appear to be a limiting factor, since for this case release is much slower as for the other drugs, and the diffusional exponent *n* that relates to the mechanism of drug release is much higher.

## CONCLUSION

We conclude that extrusion followed by injection moulding is a suitable method for preparing sustained release caplets for a range of drugs, varying in hydrophobicity. The caplet preparation procedure leads to a reduction of crystallinity for all drugs, although the reduction is smallest for the most hydrophobic drug. We also find that electrostatic attractions between matrix and drug can significantly slow down release. We show that depending on drug charge and hydrophobicity zein can show a wider variety of release characteristics.

## Electronic supplementary material

ESM 1 (DOCX 1.37 MB)

## ABBREVIATIONS

ATR-FTIR: Attenuated Total Reflection Fourier Transform Infrared Spectroscopy
DSC: Differential Scanning Calorimetry
HME: Hot-melt extrusion
HME-IM: Hot-melt extrusion-injection moulding
IM: Injection moulding
PXRD: Powder X-ray diffraction
SEM: Scanning electron microscopy
T_g_: Glass transition temperature
XRT: X-ray tomography
