# Supplementary material for: Controlled Release from Zein Matrices: Interplay of Drug Hydrophobicity and pH
Source: Pharm Res. 2015 Nov 18;33:673–85. doi: 10.1007/s11095-015-1818-8 (PMC4744255; doi:10.1007/s11095-015-1818-8)
Supplement: Supplementary file 1 — (DOCX 1.37 MB) [file 11095_2015_1818_MOESM1_ESM.docx]

Supplementary materials


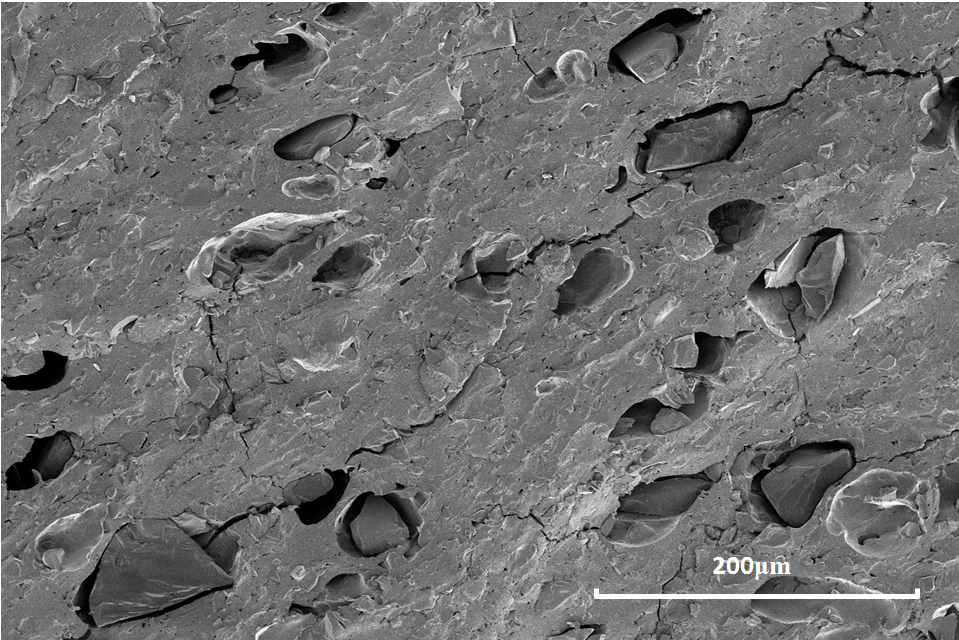


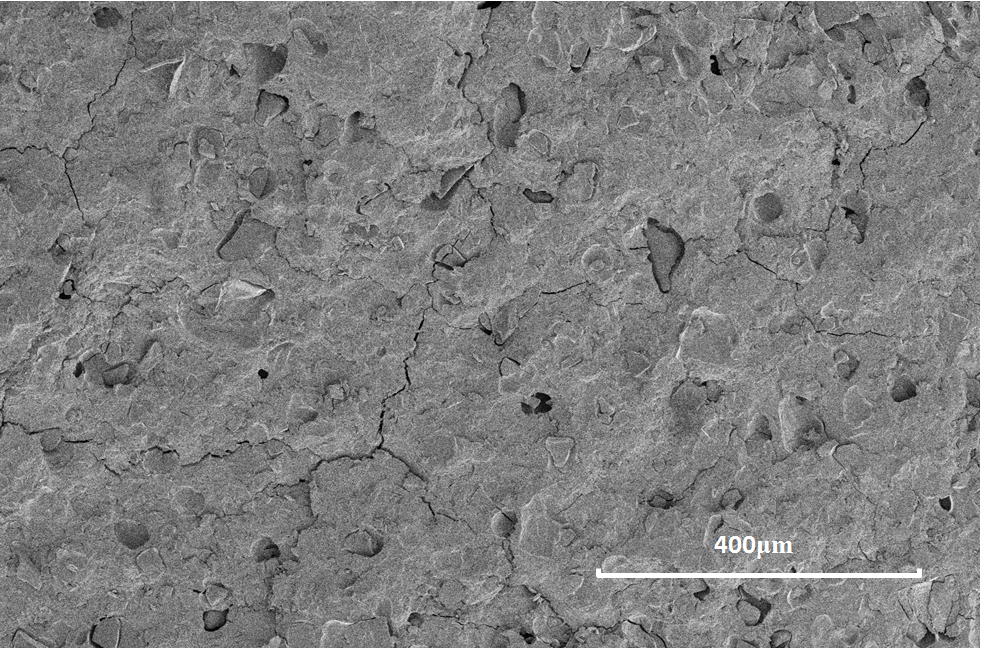


Figure 1: SEM images of cut cross sections of 22.2% loaded indomethacin caplets
